# Identification of Moldy Peanuts under Different Varieties and Moisture Content Using Hyperspectral Imaging and Data Augmentation Technologies

**DOI:** 10.3390/foods11081156

**Published:** 2022-04-16

**Authors:** Ziwei Liu, Jinbao Jiang, Mengquan Li, Deshuai Yuan, Cheng Nie, Yilin Sun, Peng Zheng

**Affiliations:** College of Geosciences and Surveying Engineering, China University of Mining and Technology (Beijing), Beijing 100083, China; liuziwei@student.cumtb.edu.cn (Z.L.); m17801150112@163.com (M.L.); yuandes@126.com (D.Y.); zhuangzhuang9905@163.com (C.N.); sun_yilin@yeah.net (Y.S.); 1610250219@student.cumtb.edu.cn (P.Z.)

**Keywords:** moldy peanut identification, moisture content, hyperspectral image, classification, data augmentation

## Abstract

Aflatoxins in moldy peanuts are seriously toxic to humans. These kernels need to be screened in the production process. Hyperspectral imaging techniques can be used to identify moldy peanuts. However, the changes in spectral information and texture information caused by the difference in moisture content in peanuts will affect the identification accuracy. To reduce and eliminate the influence of this factor, a data augmentation method based on interpolation was proposed to improve the generalization ability and robustness of the model. Firstly, the near-infrared hyperspectral images of 5 varieties, 4 classes, and 3 moisture content gradients with 39,119 kernels were collected. Then, the data augmentation method called the difference of spectral mean (DSM) was constructed. K-nearest neighbors (KNN), support vector machines (SVM), and MobileViT-xs models were used to verify the effectiveness of the data augmentation method on data with two gradients and three gradients. The experimental results show that the data augmentation can effectively reduce the influence of the difference in moisture content on the model identification accuracy. The DSM method has the highest accuracy improvement in 5 varieties of peanut datasets. In particular, the accuracy of KNN, SVM, and MobileViT-xs using the data of two gradients was improved by 3.55%, 4.42%, and 5.9%, respectively. Furthermore, this study provides a new method for improving the identification accuracy of moldy peanuts and also provides a reference basis for the screening of related foods such as corn, orange, and mango.

## 1. Introduction

Peanut (*Arachis hypogaea* L.) is an important raw material for edible oil production, which contains nutrients such as proteins, carbohydrates, lipids, and vitamins [1]. Frequent consumption of peanuts can effectively reduce the risk of cardiovascular disease [2,3]. However, peanuts are prone to mildew in a humid and muggy environment. Toxins such as aflatoxins in moldy peanuts are carcinogenic and mutagenic to humans [4,5]. Therefore, it is necessary to screen out these moldy peanuts in food production. Hyperspectral technology is often used for rapid non-destructive testing of agricultural products [6,7,8]. In previous studies, researchers have used hyperspectral technology to identify moldy peanuts [9,10,11,12]. For example, He et al. used visible-near infrared hyperspectral images to classify 150 peanuts naturally polluted by aflatoxin B1 at the particle level, and achieved a classification accuracy of 94% on the support vector machines (SVM) classifier [9]. Liu et al. used 400–1000 nm hyperspectral images to classify 2171 peanuts and constructed a band selection model for feature selection to identify healthy, moldy, and damaged peanuts. The classification accuracy of 97.66% was achieved when 10 feature bands were used in the ShuffleNet V2 model [10]. Therefore, it is feasible to carry out the identification of moldy peanuts based on hyperspectral technology.

In production, peanuts are usually exposed to the sun after harvest to remove excess moisture. According to the national standard GBT1532-2008 of China, the moisture content of sun-dried peanuts is generally less than 9%. In a humid environment, dry peanuts will absorb moisture from the environment and become moldy, and their moisture content will increase. In this way, the quality and moisture content of the same batch of peanuts will change greatly. In the study of moldy peanut identification, some researchers use oven drying or exposure to control the potential effect of moisture content on the spectrum [13,14,15]. Some researchers directly used the original sample [9,16]. The peanut moisture content in actual production, especially the moisture content of different batches or different treatment conditions, will be quite different. However, there is a lack of study on the effect of peanut moisture content on classification in the current research.

Many studies have proved that the difference in moisture content will affect the identification accuracy of objects [17,18,19]. For example, when identifying wood types, Russ et al. found that the successful recognition rate of wet wood chips was higher than that of dry wood chips [17]. Wei et al. identified the main fungi of moldy walnut and the effects of storage conditions on walnut kernels. It was found that the effect of moisture content was greater than that of temperature and relative humidity [19]. In the actual production, in addition to moisture content, there may also be impurity, germination, variety, clay, and other factors. Therefore, the scenario on the production line will be more complex than that of the training set used in model training. That is, the scenario of the test set will be more complex than that of the training set. To reduce and eliminate the influence of potential factors, data augmentation during model training is an effective way to improve the generalization ability and robustness of the model.

The common data augmentation methods mainly include four categories. One is based on geometric transformation, such as flipping, rotation, translation, scaling, clipping, and so on. For example, the Cutmix method [20] replaces a rectangular region in one image with a rectangular region of the same size in another image. Mosaic Data Augmentation [21] uses four images for random clipping, scaling, rotation, and other operations to synthesize an image, which is often used for data augmentation in target detection. The second is the method based on color transformation, including adding random noise, blur, color transformation, and so on. For example, the Cutout method [22] generates new data by randomly erasing rectangles of indefinite position and size. The third is the method based on the idea of interpolation [23,24,25,26], such as SMOTE [25] and Mixup [27]. The principle of the Mixup method is to generate new samples by multi-sample weighting. Although it has a certain effect on improving the classification accuracy, the texture features of the new samples are destroyed and the interpretability is poor. The fourth is the method based on image generation, that is, the method of generative adversarial networks (GAN) [28,29,30,31] series. From GAN [32], conditional generative adversarial networks (cGAN) [33] to deep convolutional generative adversarial networks (DCGAN) [34], stacked generative adversarial networks (StackGAN) [35], and so on, the problem of mode collapse and vanishing gradients in this kind of method has not been well addressed [36,37], which makes the training process unstable and leads to the instability of the quality of the generated data.

Moreover, for hyperspectral images, Acción et al. constructed a dual-window superpixel method to generate new data by flipping and mirroring the internal regions of the patch images [38]. Li et al. constructed a hyperspectral data augmentation method called pixel block pair [39]. Qin et al. combined the Hapke equation and a priori knowledge of hyperspectral reflection to construct a new data augmentation method for mineral analysis [40]. Haut et al. built a data augmentation method by randomly occluding the interior of image blocks [41], which is similar to the idea of Cutout. Miftahushudur et al. expanded the training data from the perspective of color temperature through spectral correction under three color temperature scenarios [42]. In addition, researchers proposed the data augmentation method by adding or subtracting the spectral mean [43] or standard deviation [44] from the original data. These methods build corresponding data augmentation methods for specific research objects and scenes. Their feasibility in peanut data needs to be verified.

In this paper, the peanut was taken as the research object, and moisture content was used as the influencing factor to study the hyperspectral data augmentation method suitable for moldy peanut identification. The specific objectives were to: (1) study the effect of moisture difference on the identification of moldy peanuts; (2) compare the identification effect of different classifiers on different varieties of peanut; (3) analyze the effect of different data augmentation methods on improving the identification accuracy.

## 2. Materials and Methods

### 2.1. Peanut Mildew Experiment

#### 2.1.1. Experimental Control of Peanut Mildew

Five varieties of peanuts including Silihong (SLH), Dabaisha (DBS), Black peanut (BLACK), Qicai (QC), and Xiaobaisha (XBS) were selected for the experiment. Each variety was about 4 kg. The peanuts were divided into healthy peanuts (HP), damaged peanuts (DP), moldy peanuts (MP), and white moldy peanuts (WP). Among them, healthy peanuts are complete and shelled healthy peanuts. In the process of peanut machine screening, damaged peanuts are the category that often needs to be screened, so damaged peanuts are also included in this experiment. Moreover, damaged peanuts include peanuts with damaged seed coats and peanuts with broken kernels.

Moldy peanuts were obtained by natural mildew of peanuts through a constant temperature and humidity incubator. The control conditions were 35 °C and 80% relative humidity. To obtain samples with different mildew degrees, the mildew process was divided into three periods, and samples were collected at the end of each period. According to the mildew degree of peanuts, the first period was from the beginning to the 14th day. The second period was from 14 to 21 days, and the third period was from 21 to 28 days. In the first period, the mold changed slowly. It changed rapidly in the second and third periods. In addition, the mildew process of peanuts in their natural state is uneven. Therefore, all the samples in the incubator were fully shaken every two days in all experimental periods, so that all peanut kernels were infected by mold.

In addition to *Aspergillus flavus*, peanuts may also be infected by other molds to form black or white mildew spots. In our previous experiments, we found that peanuts that were soaked in water were prone to produce white mold. To obtain moldy peanuts of this character, the peanuts were put into a sealed plastic box and sprayed with the proper amount of water every two days. After the same period, the white moldy peanuts were obtained.

The color and texture features of moldy peanuts are different from those of healthy peanuts. To confirm that the cultured moldy peanuts produced aflatoxin, we commissioned the Agricultural Products Quality Supervision, Inspection, and Test Center of China Agricultural University to detect the aflatoxin content of moldy peanuts and white moldy peanuts. According to commission regulation, NO 165/2010 released by the European Union, the sum of aflatoxin B1, aflatoxin B2, aflatoxin G1, and aflatoxin G2 of peanuts directly for human consumption or as food ingredients cannot exceed 4 ug/kg. The results showed that the toxin content of all moldy peanuts had greatly exceeded the threshold. Therefore, it is considered that the peanut samples used in the experiment are accurate.

#### 2.1.2. Controlling the Moisture Content Gradient of Peanut

To control the moisture content gradient of peanuts, some samples were used to bake at 70 °C and weighed every 2 h to observe the weight changes of peanuts. It was found that the moisture content of peanuts decreased to about half of the original moisture content after baking for 4 h. Therefore, the untreated peanut samples were taken as the first moisture content gradient. The samples dried at 70 °C for 4 h as the second moisture content gradient, and the samples dried at 70 °C to constant weight as the third moisture content gradient. The three gradients were named G1, G2, and G3, respectively. The following Formula (1) was used to calculate the moisture content (wet basis) of each gradient peanut:

(1) $$M_{C}=\frac{W_{1}-W_{2}}{W_{1}-W_{C}}\times 100\%$$

where $M_{C}$ is the moisture content of peanut, $W_{1}$ is the weight of utensil and samples before baking, $W_{2}$ is the weight of utensil and samples after baking, and $W_{C}$ is the weight of utensil.

### 2.2. Data Acquisition and Preprocessing

**Figure 1:**
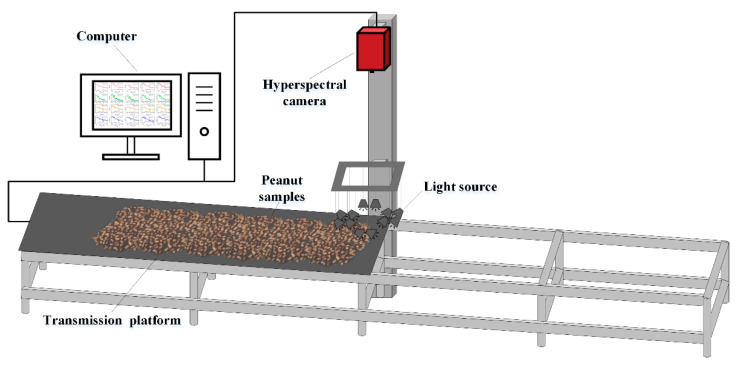
Hyperspectral image acquisition system.

A hyperspectral image acquisition system was set up to collect the hyperspectral images of peanuts. The equipment schematic diagram is shown in Figure 1. The spectrometer is HySpex SWIR-384 (Norsk Elektro Optikk AS, Norway) with the spectral range of 930–2500 nm. The spectral channel is 288 bands and the spectral sampling is 5.45 nm. The light source is composed of twelve halogen lamps (50 watts each). A strip whiteboard was placed at the beginning of the transmission platform for spectral reflection correction. During the scanning, the peanuts were placed on the transmission platform with a black background. The movement of the transmission platform was controlled by a computer, and the moving speed of the platform was the same as the scanning rate of the spectrometer.

According to the mildew period, peanut variety, and moisture content gradient, the corresponding hyperspectral images were obtained by the hyperspectral image acquisition system. The acquired data needs to be divided into a training set and a test set.

In the training set, for each variety, nine images of HP were collected with three images for each gradient. Six images of DP were collected with two images for each gradient. WP was the same as DP. Eighteen images of MP were collected with two images for each gradient and each period. A total of 195 images of the training set were obtained.

In the test set, for each variety, three mixed class images were collected for each variety and each period. In addition, three images of all varieties, classes, and gradients were collected. A total of 54 images were collected in the test set. Finally, a total of 249 images were obtained.

For each image, 162 peanuts were placed in the training set, 144 peanuts in the test set, and 120 peanuts in all varieties. Finally, a total number of 39,119 peanut kernels were obtained, including 7819 BLACK images, 7788 DBS images, 7820 QC images, 7869 SLH images, and 7823 XBS images.

The detailed data information is shown in Table 1. Because the seed coat of healthy peanuts may fall off after baking, the category of healthy peanuts becomes damaged peanuts. In data processing, the peeled kernels in healthy peanuts were set as damaged samples. This did not occur in the samples of moldy peanuts and white moldy peanuts. The format of the obtained images was converted from digital number to radiance, and then a region of interest was selected in the white plate of the image for spectral correction. The correction formula is as follows:

(2) $$R=\frac{O-D}{W-D}$$

where R is the corrected spectral reflectance, O is the original radiance, D is the spectral reflectance of dark plate, and W is the reflectance of white plate. Then the final reflectance data was obtained.

A feature band (1901 nm) was selected as the original mask for distinguishing peanut kernels and background. Firstly, the appropriate threshold was selected to distinguish the background and peanut kernels. The background pixels in the mask were set to 0, and the kernel pixels were set to different values according to the variety and class. Then, the noise and non-peanut pixel masks were set to zero by manual labeling. At the same time, a few adjacent kernels were also separated. All the hyperspectral images of peanut kernels were extracted by a watershed segmentation algorithm using a mask.

Because this paper used two types of the classification model, one was based on point spectral data, and another was based on hyperspectral image data. Therefore, for the method based on point spectrum, the average spectrum of each kernel was calculated as a training unit, and the corresponding label was set according to the type of peanut kernel. In this way, a peanut data set covering five varieties, four classes, three moisture content gradients, and two data types was completed.

### 2.3. Data Augmentation

#### 2.3.1. Constructed Data Augmentation Method

To improve the identification accuracy of the classification model, a data augmentation method based on data interpolation was constructed. Compared with individual samples, the feature information of group samples may be more important. For the hyperspectral dataset K:

(3) $$K=k_{1},\;k_{2},\;\ldots ,k_{n-1},\;k_{n}$$

where k is the hyperspectral image cube of peanut kernels and n is the total number of samples.

Firstly, the average spectrum of each kernel was calculated, and the corresponding spectral dataset SP was obtained.

(4) $$SP=\left\{\begin{array}{c} S1_{1},\;S1_{2},\;\ldots ,S1_{a-1},\;S1_{a}\\ \ldots ,\\ Sc_{1},\;Sc_{2},\;\ldots ,Sc_{b-1},\;Sc_{b} \end{array}\right.$$

where S is the average spectrum of each kernel, c is the number of peanut classes, and a, b are the sample number of each class of peanut. A feature band was selected to sort the sample reflectance values of each class. Then, the data of this class was divided into two parts with the median as the boundary, and the average spectrum of each part was calculated for generating the spectral difference SD of the two parts. The sd was used as the baseline of the original spectral offset. The generated spectral data NS_spectral can be expressed as:

(5) NS_spectral=S+λ×SD

where λ is a parameter used to adjust the offset of the original data. In this way, the data was interpolated by offsetting the original spectrum.

For kernel hyperspectral image data, the augmented data NS_image can be expressed as:

(6) $$NS\_image=rotate\left(K+\lambda \times SD\right)$$

The image rotation was added on the basis of spectral shift. Moreover, the maximum and minimum values of the reflectance of the extended data generally exceed the original reflectance. That is, it will produce some outliers. These outliers can fill the missing moisture content gradient data to some extent and increase the robustness of the model. In this way, the purpose of improving the generalization ability of the model was achieved. This method expanded the data through the difference in the spectral mean, so it was named the DSM method.

#### 2.3.2. Compared Data Augmentation Methods

Some data augmentation methods showed good results in the original paper, but they were not suitable for the experimental data and subjects of this study. Therefore, according to the characteristics of peanut hyperspectral data, the commonly used methods and frontier methods were compared to verify their effects.

Specifically, the principle of the original random erasing method [41] was to randomly erase a small rectangular area in the image. For the point spectral data, we modified it to randomly mask the spectrum of 10 bands, that is, randomly set 10 continuous bands to zero. In the random noise method, a random noise of [−0.01, 0.01] of the same length as the spectral data was added to the original spectrum to generate new data. The original Mixup method fused two images by weight to generate new data. Due to the point, spectral data does not contain texture information, it cannot be directly used in point spectral data. Here, we extended the implementation of this method to the weighted summation of two spectra and called it the two sample weighting (TSW) method. The formula is as follows:

(7) $$TSW=\theta \times S_{i}+\left(1-\theta \right)\times S_{j}$$

where i, j are two spectral samples, $\theta \in \left[0,\;1\right]$. On the whole, for the classification methods based on spectrum, the original data (None), random erasing (Erasing), random noise (Noise), TSW, and DSM were compared.

For the image-based classification method, the original data (None), random erasing (Erasing), random noise (Noise), rotation (Rotation), and DSM were compared. TSW method was not adopted for the reason that the texture information of the data will be destroyed and the generated data lacks interpretation. Similarly, a random erase area of 5×5 was set in the random erasing method. In the random noise method, the random noise between [−0.01, 0.01] of the same shape as the hyperspectral image cube was generated. Then, it was added to the original spectral cube to get the augmented data. In the rotation method, under twice the sample size, the original image was rotated 90 degrees clockwise to generate augmented data. Under four times the sample size, the rotation of 90 degrees, 180 degrees, and 270 degrees was set.

### 2.4. Classification Model

Three classification models, KNN [45], SVM [46] and MobileViT [47], were used for classification. Among them, KNN and SVM are classical pixel-based classifiers, which are widely used in classification research. The basic principle of the KNN algorithm is to judge the class of the input sample according to the class of the K points closest to the input sample. It is a supervised classification model that runs fast under a large amount of data. The training time of KNN is short, and it is not sensitive to abnormal values. The gradient data that do not participate in the training will contain many abnormal values. Given this feature, KNN was selected for classification.

The basic principle of SVM is to find a hyperplane in the feature space that can separate all data samples and minimize the distance between the data in the training set and the hyperplane. It is one of the most widely used classifiers. In many classification tasks, there is often a case of linear inseparability. Therefore, SVM based on kernel functions such as radial basis function, n-order polynomials, and sigmoid is used to solve this problem. The kernel function used in this paper is the radial basis function.

**Figure 2:**
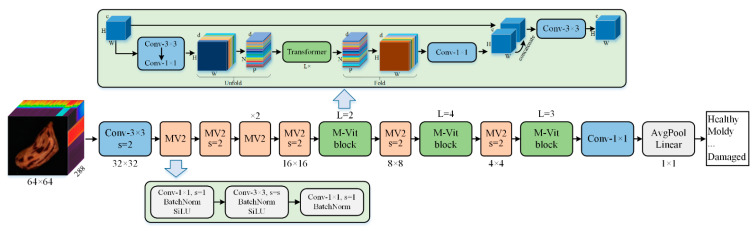
Model structure of MobileViT-xs. MV2: MobileNetv2 block, s: stride, M-Vit block: MobileViT block, H: height; W: width; c: channels; P: the number of pixels in the patch; N: the number of patches; L: the number of transformer blocks.

MobileViT is a lightweight deep learning model based on a transformer [48], which shows good performance in image classification, semantic segmentation, and object detection. The model includes three architectures, namely, MobileViT-s, MobileViT-xs, and MobileViT-xxs, with different numbers of parameters. According to the results of the original research, MobileViT-xs was selected as the classification model of this study by weighing the parameters and accuracy. The model structure is shown in Figure 2. The model is mainly composed of the MobileNetv2 block and MobileViT block, and the detailed description of the model can refer to the original literature [47].

### 2.5. Statistical Analysis and Experimental platform

Pearson correlation coefficient was used to determine the relationship between two gradient results and all gradient results. The SciPy package in Python was used to calculate the Pearson correlation coefficient, where the parameters p and r reflect the significance level and correlation, respectively. Significant differences were considered when p<0.05. The positive or negative value of r reflected the positive or negative correlation. Difference analysis was used to analyze the differences of different gradient results, as well as to analyze the effect of data augmentation methods.

All the experiments were run on Intel Core i7-12700 (2.10 GHz), 64GB RAM and NVIDIA RTX3060 GPU with 12 GB memory. Python 3.7.1 was used to implement all program code. The functions in the Sckit-learn package were used to realize the KNN and SVM algorithms. The MobileViT-xs model was implemented by the deep learning framework PyTorch 1.10, and CUDA Toolkit 11.3 was used to accelerate the processing. More code details were posted at https://github.com/mepleleo/DA_peanut (accessed on 1 April 2022).

## 3. Results and Discussion

### 3.1. Experimental Configuration

In this experiment, the peanut data of three moisture content gradients were obtained. Two groups of experiments were carried out. In experiment 1, the G1 and G2 gradient data in the training set were used to train the model, and the test set containing all gradient data was used to test the model. In experiment 2, the G1, G2 and G3 gradient data in the training set were used to train the model, and the test set containing all gradient data was used to test the model. In order to control the experimental variables, the same number of training samples as in experiment 1 were randomly selected from all gradient data.

For the parameters of the model, the grid search algorithm was used to determine the optimal hyperparameters of KNN (neighbors) and SVM (C, gamma) models. In KNN model, the distance measurement method was set to ‘minkowski’ distance. Other parameters of the models were set to default values. When training MobileViT-xs, the original images were padded to 64×64×288, and the padding value was zero. The batch size was set to 128 and epochs were set to 50. The Adam optimizer was used and the initial learning rate was 0.001 with the decay rate of 0.2 times per 10 epochs.

### 3.2. Analysis of Spectral Characteristics and Moisture Content

#### 3.2.1. Moisture Content and Spectral Reflectance Characteristics

**Figure 3:**
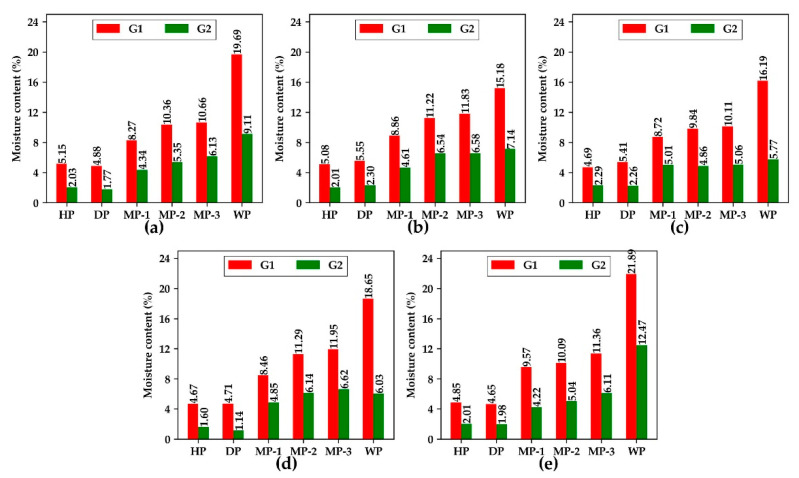
Moisture content of different varieties of peanut. (**a**) BLACK, (**b**) DBS, (**c**) QC, (**d**) SLH, (**e**) XBS. HP, healthy peanuts; DP, damaged peanuts; MP-(1-3), moldy peanuts in three periods; WP, white moldy peanuts; G1, the first moisture content gradient; G2, the second moisture content gradient.

The moisture content of peanuts is shown in Figure 3. The moisture content of the G3 gradient was zero. Therefore, it was not plotted in the figure. The range of peanut moisture content was 0–21.89%. Among them, the moisture content range of HP and DP was 0–5.15% and 0–5.55% respectively. The range of MP and WP was 0–11.95% and 0–21.89% respectively. The moisture content of MP was higher than that of HP. At 80% humidity, peanuts absorb moisture from the environment, resulting in an increase in moisture content. WP shows the highest moisture content for the reason that peanuts absorb more water after spraying water. After baking for 4 h, the average moisture content of peanuts decreases to about half of the original sample.

**Figure 4:**
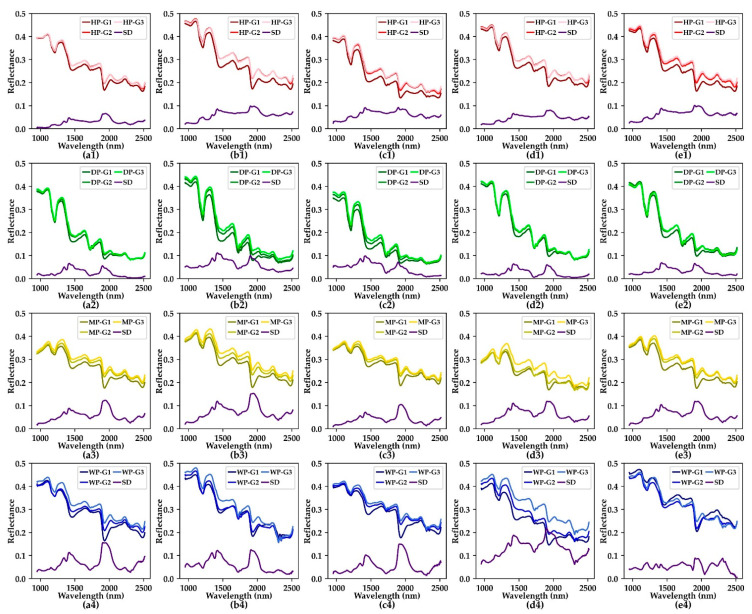
Average spectrum of each moisture content gradient for all varieties and classes. (**a1**–**a4**) BLACK, (**b1**–**b4**) DBS, (**c1**–**c4**) QC, (**d1**–**d4**) SLH, (**e1**–**e4**) XBS. HP, healthy peanuts; DP, damaged peanuts; MP, moldy peanuts; WP, white moldy peanuts; G1, the first moisture content gradient; G2, the second moisture content gradient; G3, the third moisture content gradient; SD, spectral difference.

Figure 4 shows the average spectrum of each moisture content gradient for all varieties and classes. As can be seen from the figure, the reflectance increases with the decrease of moisture content. From the purple spectral difference curve, it can be seen that the reflectance changes differently at different wavelengths. A common feature is that the reflectance of peanuts changes most near 1910 nm and 1420 nm, which is related to the first-order frequency doubling stretching vibration of O-H [49,50]. In terms of spectral reflection characteristics, there are obvious spectral absorption valleys at 1209 nm, 1471 nm, 1727 nm, 1934 nm and 2484 nm. These feature bands mentioned above can be used as reference bands for spectral feature extraction.

#### 3.2.2. Data Distribution after Data Augmentation

**Figure 5:**
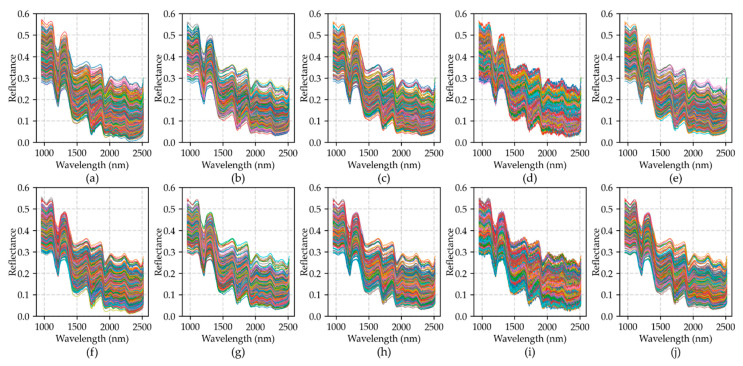
Data distribution after data augmentation for SLH damaged peanut class. (**a**–**e**) G1 and G2 data using None, Erasing, Noise, TSW and DSM respectively; (**f**–**j**) all gradient data using None, Erasing, Noise, TSW and DSM respectively.

Figure 5 takes SLH DP as an example to show the spectral distribution after twice augmentation by different data augmentation methods. Among them, Figure 5a–e takes G1 and G2 as the training data. Because the sample reflectance of G3 was higher than that of G1 and G2, the λ of DSM was set as [−0.2, 0.8] to shift the sample distribution to high reflectance. Figure 5f–j is the distribution of all gradient data after augmentation. Because both the training set and the test set contain three gradient moisture content data, the λ of DSM was set as [−0.5, 0.5] make the sample distribution more uniform while interpolating.

In this experiment, the function of data augmentation was to make up for the lack of moisture gradient data. In Figure 5e, the spectra with high reflectance increased after the expansion of DSM method, which is closer to the spectral distribution of Figure 5f. This can promote the improvement of identification accuracy. At the same time, a small number of outliers can improve the generalization ability of the model, so that the model has a better recognition ability when identifying unknown samples. The Erasing method is to erase a spectrum on the basis of the original sample. Therefore, the data distribution is similar to the original sample. In the Noise method, the augmented data has more spectral fluctuations than the original sample. The TSW method interpolates between the maximum spectral reflectance and the minimum reflectance of the original spectrum.

### 3.3. Classification Results and Analysis

In order to make the experimental results more accurate, all the classification results were obtained through an average of five independent experiments. In the KNN and SVM algorithms, the accuracy results of the original data, twice the sample size and four times the sample size were compared respectively. The training process of MobileViT-xs is time-consuming. In order to save the time cost, a comparison was made only under the data of twice the sample size. In this experiment, each variety is an independent data set, and a data augmentation method may not achieve the highest accuracy on all data sets. Therefore, the average improved accuracy (AIA) of each method on five varieties of peanut datasets was calculated. In the classification result tables, the data amount of the original training sample was expressed as N. The sample size of the training set including original data and once augmentation data was expressed as 2N. Similarly, the sample size after three times augmentation was expressed as 4N.

#### 3.3.1. Classification Results of Two Gradient Data

Table 2 shows the classification results of KNN using G1 and G2 data. Obviously, the DSM method constructed in this paper achieved the highest classification accuracy under the sample size of 2N and 4N. Compared with the classification accuracy of the original sample, the identification accuracy was improved by 1.02% to 3.46% under the 2N sample size and 1.87% to 4.55% under the 4N sample size. The classification effect on QC and SLH was the best, and both of them were more than 90%. Compared with the TSW method, the DSM method takes into account the spectral features of the whole sample, while the TSW method only considers the spectral features of the pairwise samples. The classification results illustrate that the method considering global sample features can achieve higher identification accuracy than the method considering independent sample feature. The accuracy of Erasing method on all varieties of peanuts tended to be zero, which indicates that the method is not effective for KNN classifier. The AIA of other methods was not as good as the constructed method.

Table 3 shows the classification results of SVM using G1 and G2 data. Similarly, the DSM method improved the accuracy by 0.74% to 6.1% under the 2N sample size and 0.98% to 8.64% under the 4N sample size, which also illustrates the effectiveness of the method. Compared with other methods, the data generated by DMS had a better effect on improving the classification accuracy. The classification accuracy of SVM was better than KNN in BLACK, DBS and QC, but on the contrary in SLH and XBS.

It should be noted that the Erasing method and the Noise method had a negative effect on the identification accuracy under the 2N sample size. The AIA of Erasing method and Noise method decreased by 0.63% and 0.11% respectively. The Erasing method was still negative under the 4N sample size. Similarly, in related research on hyperspectral image classification [38,43], we also found that some data augmentation methods lead to a decrease in classification accuracy. This illustrates that only the data augmentation method suitable for the classifier can improve the identification accuracy of the model. On the one hand, the quality of the data produced by data augmentation may not be as good as the original data. On the other hand, it is caused by the insufficient feature extraction performance of the classifier.

The classification results of the MobileViT-xs model are shown in Table 4. The DSM method improved the accuracy by 4.06% to 7.6% under the 2N sample size. Compared with KNN and SVM, the deep learning model MobileViT-xs gained the most from data augmentation, which is consistent with related research results [31]. At the same time, the classification accuracy of all varieties was more than 92%. It demonstrates that the ability of the model to identify outliers was greatly enhanced by data augmentation. Other data augmentation methods also improved the accuracy to varying degrees. This was mainly due to the strong fitting ability and generalization ability of the deep learning model. In terms of standard deviation results, the standard deviation of MobileViT-xs model was slightly larger than that of KNN and SVM. Although the accuracy in the training set was almost the same, the slight change of model parameters will lead to the fluctuation of classification accuracy in the test set. However, this cannot deny the recognition effect of the model. Different from the SVM classifier, the methods of Erasing and Noise demonstrated an active role in MobileViT-xs model, and the average improvement in five varieties was 4.63% and 3.49% respectively.

Compared with the related studies on moldy peanut identification, previous studies generally only carried out identification in fewer varieties and simple scenarios. For example, Liu et al. carried out a study on the identification of single variety and three classes of moldy peanuts [15]. Sun et al. conducted a moldy peanut identification study on four varieties, but only two varieties, moldy and healthy, were considered [51]. Qi et al. carried out a study on the identification of moldy peanuts on two varieties and two classes [14]. In this study, five varieties and four classes of peanuts were used in the experiment, and the effect of moisture content was also considered. Overall, the accuracy of 4N sample size is higher than that of 2N sample size. After data augmentation, the classification accuracy of the best model on the five varieties of peanut was more than 90%. The classification accuracy on QC and SLH is satisfactory. Especially on the MobileViT-xs model, their classification accuracy after data augmentation all exceeded 96%. Although the classification accuracy of BLACK, DBS and XBS on all models was more than 81%, there is potential for accuracy improvement. Therefore, it is necessary to carry out research on the identification of moldy peanuts under all gradients.

#### 3.3.2. Classification Results of All Gradient Data

Table 5 shows the classification results of KNN using all gradient data. Compared with Table 2, the accuracy improvements of BLACK, DBS, QC, SLH and XBS without data augmentation were 3.1, 1.53, 0.06, 0.64 and 5.07 respectively. The classification accuracy of all gradient training data was higher than that of two gradient data. This illustrates that the spectral changes caused by the difference of peanut moisture content will affect the identification accuracy. Comparing Table 2 and Table 5, the results of the two gradient data using the DSM method and 4 times data augmentation have greatly exceeded the results of all gradient data without data augmentation. This indicates that the data augmentation method constructed in this paper can effectively solve the problem of the decline of classification accuracy caused by the lack of moisture content information.

Moreover, in the training results of all gradient data, the DSM method improved the accuracy by 1% to 2.18% under the 2N sample size and 2.23% to 3.44% under the 4N sample size, which proves that the DSM method can further improve the classification accuracy on the basis of the recognition accuracy of all gradient data. Other methods were less effective than the proposed method.

In Table 6, the classification accuracy of all gradient data on SVM classifier was higher than that of two gradients (Table 3). This fully demonstrates that the difference of moisture content will affect the identification accuracy of peanuts. Therefore, it can be inferred that the richer the moisture content information of the training set is, the higher the classification accuracy is. Comparing Table 3 and Table 6, it can be found that data augmentation can only weaken the influence of moisture content on accuracy. After data augmentation, the model accuracy using all gradient data greatly exceeded two gradients. Therefore, it is necessary to obtain sufficient moisture content information to fundamentally improve the model accuracy.

Similarly, the classification results of different data augmentation methods tend to be consistent in the same classifier. Especially under the sample size of 4N, the average improvement of DSM on each variety of peanut was 2.89%. Although the classification accuracy of DSM method was slightly lower than that of TSW method on QC. However, it was better than TSW method in other peanut datasets.

It can be seen from Table 7 that all the data augmentation methods played a positive role in improving the accuracy of MobileViT-xs model. Compared with Table 4, the accuracy improvements of BLACK, DBS, QC, SLH and XBS without data augmentation were 4.1, 3.8, 1.7, 1.73 and 0.64 respectively. This further indicates that the moisture content has a great influence on the classification accuracy. Compared with KNN and SVM, MobileViT-xs model can still achieve higher identification accuracy even without data augmentation. The identification accuracy of all peanut varieties except DBS was more than 96%. Although DBS achieved the accuracy of 92.57% after data augmentation, the result was still not good enough. Therefore, a larger amount of data or detailed parameter adjustment may be needed to further improve its accuracy.

In addition, the identification accuracy of the MobileViT-xs model was only verified on the 2N sample size. If more training data are used, the model should be able to achieve higher identification accuracy. The model had achieved high accuracy under all gradient data. Therefore, the accuracy improvement of the augmented data was not as large as the two gradient training data.

In the related research on moldy peanut identification, Qi et al. used a feature extraction method based on continuous wavelet transform to identify 547 peanuts and achieved a classification accuracy of more than 96.19% [12]. Qiao et al. used variance analysis and nonparametric weighted feature pre-extraction to identify 189 peanuts and achieved a pixel classification accuracy of more than 94.2% on the SVM model [13]. It should be noted that, in addition to the above mentioned, many studies [9,11,14,51] only used no more than 500 peanut samples for research, while the number of samples used in this study was close to 40,000. Although some studies achieved higher classification accuracy, sufficient samples make our study more persuasive and representative. In addition, due to the information differences of different peanut varieties, the classification accuracy of the model is closely related to the complexity of the dataset and the recognition ability of the classifier.

#### 3.3.3. Comparison and Analysis of Different Gradient Results

**Figure 6:**
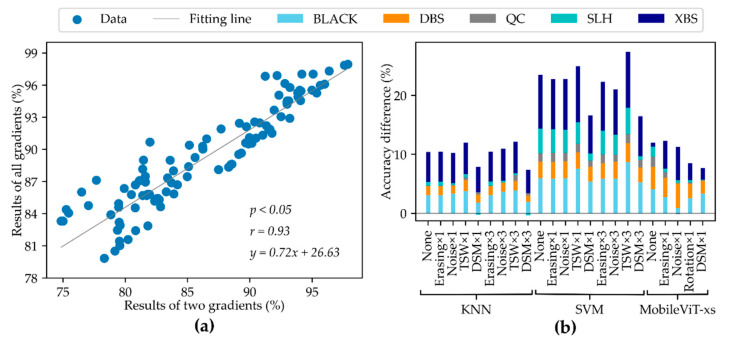
Comparison of different gradient results. (**a**) correlation of two gradient results and all gradient results, (**b**) difference between two gradient results and all gradient results. The results of two gradients indicate the results of G1 and G2. The results of all gradients indicate the results of G1, G2, and G3. BLACK, Black peanut; DBS, Dabaisha; QC, Qicai; SLH, Silihong; XBS, Xiaobaisha; None, original data; Erasing, random erasing; Noise, random noise; TSW, two sample weighting; DSM, difference of spectral mean; Rotation, rotation; KNN, K-nearest neighbors, SVM, support vector machines.

To analyze the results in Table 2, Table 3, Table 4, Table 5, Table 6 and Table 7 intuitively, correlation analysis and difference analysis were carried out. Figure 6a shows the correlation between two gradient results and all gradient results. The p-value between two gradient results and all gradient results was less than 0.05, proving that there is a significant difference between them. That is, the difference in moisture content can significantly affect the peanut identification accuracy. At the same time, it reflected a positive correlation, that is, the identification accuracy of all gradients was generally higher than that of two gradients. Figure 6b shows the difference between two gradient results and all gradient results for different methods and varieties. For different classification models, the difference in training data had the greatest impact on the SVM model. Meanwhile, the proposed DSM method demonstrated the best performance in resisting the influence of data changes.

In addition to the moisture content factor, various interference factors should be enriched as much as possible in the training process to improve the robustness and generalization ability of the model. These factors also include impurities, germination, clay, and so on. Apart from data augmentation, better performance can also be achieved through detailed parameter adjustment and model structure optimization.

### 3.4. Visualization of Results

**Figure 7:**
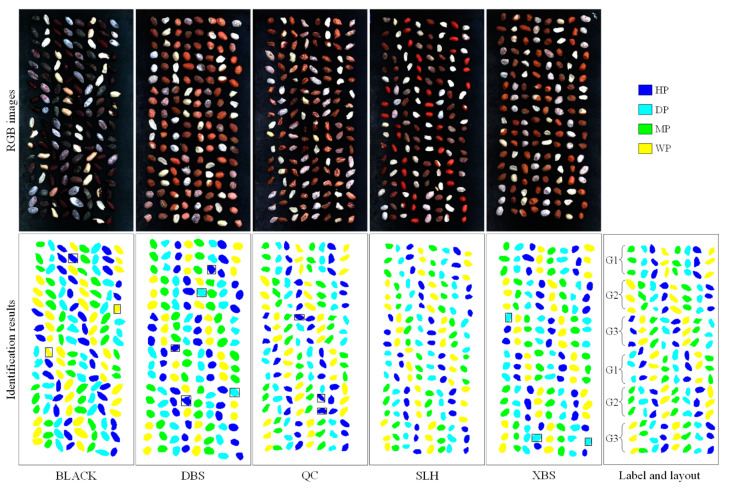
Visualization of classification results. BLACK, Black peanut; DBS, Dabaisha; QC, Qicai; SLH, Silihong; XBS, Xiaobaisha; HP, healthy peanuts; DP, damaged peanuts; MP, moldy peanuts; WP, white moldy peanuts; G1, the first moisture content gradient; G2, the second moisture content gradient; G3, the third moisture content gradient.

The classification accuracy based on MobileViT-xs and DSM is better than that of KNN and SVM, so this model was used to visualize the results. Figure 7 shows the identification results of the test images of each variety. Misidentified kernels were marked with black boxes. The peanuts were repeated twice in the order of G1, G2, and G3 with 3 rows of samples per gradient. As can be seen from the result images, the main classes of misidentification were HP and DP. The main type of misidentification was that the DP was mistakenly identified as HP. This is due to the textures of mildly damaged DP and HP being similar except for the damaged parts. Therefore, it is recommended to increase the sample size of mildly damaged peanuts to further improve the identification accuracy.

## 4. Conclusions

In this study, five varieties of peanut were taken as the research object, and the moisture content was taken as the influencing factor. Their impact on spectral reflectance and identification accuracy was analyzed. To reduce and eliminate this influence, a data augmentation method DSM based on interpolation was proposed. The method was compared with the frontier data augmentation methods. The experimental results show that the more moisture content information of the peanut dataset, the higher the classification accuracy. Data augmentation can only weaken the impact of the lack of moisture content information. It is necessary to provide more moisture content information to achieve higher classification accuracy. Among KNN, SVM, and MobileViT-xs, the image-based classification model MobileViT-xs showed the best recognition effect on five varieties of peanuts, and achieved a classification accuracy of more than 90% after data augmentation. The accuracy improvement of the DSM method was better than that of other methods in both two gradient data and all gradient data. Especially under all gradient data, the MobileViT-xs model achieved over 96% classification accuracy on four peanut varieties. Furthermore, this study has reference significance for the identification of soybean, apple, mango, and other agricultural products based on hyperspectral. Based on this research, future work will further improve the accuracy of the model in more complex scenes and more influencing factors.

## Figures and Tables

**Table 1:** Overview of experimental data sets.

	Peanut Classes	Gradient	BLACK	DBS	QC	SLH	XBS
Training set	HP	G1	491	488	482	504	492
		G2	483	475	471	494	483
		G3	476	447	470	491	481
	DP	G1	316	322	328	323	318
		G2	324	323	338	325	322
		G3	329	360	333	335	327
	MP-1	G1	324	324	324	324	324
		G2	324	324	324	324	324
		G3	324	324	322	324	324
	MP-2	G1	324	324	324	323	324
		G2	324	324	324	323	324
		G3	324	324	324	323	324
	MP-3	G1	324	315	324	324	324
		G2	324	315	324	324	324
		G3	324	315	324	324	324
	WP	G1	324	324	324	324	324
		G2	324	324	324	324	324
		G3	324	324	324	324	324
Test set	HP	G1/G2/G3	378	378	378	378	378
	DP	G1/G2/G3	378	378	378	378	378
	MP-(1-3)	G1/G2/G3	378	378	378	378	378
	WP	G1/G2/G3	378	378	378	378	378
Total			7819	7788	7820	7869	7823

BLACK, Black peanut; DBS, Dabaisha; QC, Qicai; SLH, Silihong; XBS, Xiaobaisha; HP, healthy peanuts; DP, damaged peanuts; MP-(1-3), moldy peanuts in three periods; WP, white moldy peanuts; G1, the first moisture content gradient; G2, the second moisture content gradient; G3, the third moisture content gradient.

**Table 2:** Results of KNN training using G1 and G2 data.

Sample Size	DA	BLACK (%)	DBS (%)	QC (%)	SLH (%)	XBS (%)	AIA (%)
N	None	79.37 ± 0	78.31 ± 0	88.29 ± 0	89.88 ± 0	78.84 ± 0	/
2N	Erasing × 1	79.37 ± 0	78.31 ± 0	88.29 ± 0	89.88 ± 0	78.84 ± 0	0
	Noise × 1	79.58 ± 0.08	79.17 ± 0.04	88.48 ± 0.11	91.03 ± 0.05	79.46 ± 0.08	0.61
	TSW × 1	80.79 ± 0.42	79.53 ± 0.1	89.1 ± 0.33	90.49 ± 0.38	80.49 ± 0.49	1.14
	DSM × 1	82.83 ± 0.28	80.22 ± 0.31	89.31 ± 0.47	91.77 ± 0.29	81.39 ± 0.69	2.17
4N	Erasing × 3	79.38 ± 0.02	78.31 ± 0	88.3 ± 0.03	89.88 ± 0	78.84 ± 0	0
	Noise × 3	79.51 ± 0.1	79.56 ± 0.15	88.56 ± 0.11	91.6 ± 0.09	79.46 ± 0.13	0.8
	TSW × 3	81.93 ± 0.18	80.83 ± 0.25	89.65 ± 0.36	91.52 ± 0.33	81.61 ± 0.53	2.17
	DSM × 3	83.89 ± 0.27	81.81 ± 0.32	90.16 ± 0.4	93.2 ± 0.25	83.39 ± 0.31	3.55

N, the data amount of the original training sample; 2N, the sample size after once augmentation; 4N, the sample size after three times augmentation; DA, data augmentation; None, original data; Erasing, random erasing; Noise, random noise; TSW, two sample weighting; DSM, difference of spectral mean; BLACK, Black peanut; DBS, Dabaisha; QC, Qicai; SLH, Silihong; XBS, Xiaobaisha; AIA, the average improved accuracy.

**Table 3:** Results of SVM training using G1 and G2 data.

Sample Size	DA Method	BLACK (%)	DBS (%)	QC (%)	SLH (%)	XBS (%)	AIA (%)
N	None	81.15 ± 0	82.61 ± 0	93.12 ± 0	83.86 ± 0	75.26 ± 0	/
2N	Erasing × 1	80.81 ± 0.03	82.42 ± 0.05	92.99 ± 0.04	81.77 ± 0.05	74.84 ± 0.05	−0.63
	Noise × 1	81.11 ± 0.16	82.5 ± 0.1	92.98 ± 0.11	83.4 ± 0.16	75.45 ± 0.22	−0.11
	TSW × 1	81.45 ± 0.44	83.29 ± 0.26	93.83 ± 0.26	85.6 ± 0.26	76.49 ± 0.31	0.93
	DSM × 1	83.6 ± 0.43	84.18 ± 0.33	93.86 ± 0.47	89.96 ± 0.52	79.91 ± 0.32	3.1
4N	Erasing × 3	80.82 ± 0.04	82.72 ± 0.07	93.0 ± 0.08	81.71 ± 0.03	75.0 ± 0.06	−0.55
	Noise × 3	81.6 ± 0.1	82.35 ± 0.13	92.99 ± 0.08	85.08 ± 0.16	77.04 ± 0.2	0.61
	TSW × 3	81.98 ± 0.42	83.7 ± 0.21	94.0 ± 0.41	86.52 ± 0.46	77.67 ± 0.68	1.57
	DSM × 3	85.14 ± 0.17	84.97 ± 0.36	94.1 ± 0.22	92.5 ± 0.55	81.39 ± 0.72	4.42

N, the data amount of the original training sample; 2N, the sample size after once augmentation; 4N, the sample size after three times augmentation; DA, data augmentation; None, original data; Erasing, random erasing; Noise, random noise; TSW, two sample weighting; DSM, difference of spectral mean; BLACK, Black peanut; DBS, Dabaisha; QC, Qicai; SLH, Silihong; XBS, Xiaobaisha; AIA, the average improved accuracy.

**Table 4:** Results of MobileViT-xs training using G1 and G2 data.

Sample Size	DA Method	BLACK (%)	DBS (%)	QC (%)	SLH (%)	XBS (%)	AIA (%)
N	None	86.2 ± 2.67	86.23 ± 1.41	90.78 ± 2.76	91.94 ± 1.77	87.48 ± 1.63	/
2N	Erasing × 1	92.33 ± 0.85	89.15 ± 0.74	96.36 ± 0.68	94.98 ± 0.32	92.18 ± 1.46	4.47
	Noise × 1	91.32 ± 0.9	87.68 ± 1.36	95.36 ± 1.99	94.06 ± 1.04	91.23 ± 1.76	3.4
	Rotation × 1	93.21 ± 0.6	89.72 ± 1.35	97.59 ± 0.29	95.65 ± 0.64	94.17 ± 0.96	5.54
	DSM × 1	92.81 ± 0.89	90.38 ± 1.61	97.87 ± 0.58	96.0 ± 0.4	95.08 ± 1.58	5.9

N, the data amount of the original training sample; 2N, the sample size after once augmentation; DA, data augmentation; None, original data; Erasing, random erasing; Noise, random noise; Rotation, rotation; DSM, difference of spectral mean; BLACK, Black peanut; DBS, Dabaisha; QC, Qicai; SLH, Silihong; XBS, Xiaobaisha; AIA, the average improved accuracy.

**Table 5:** Results of KNN training using all gradient data.

Sample Size	DA	BLACK (%)	DBS (%)	QC (%)	SLH (%)	XBS (%)	AIA (%)
N	None	82.47 ± 0.39	79.84 ± 0.13	88.35 ± 0.19	90.52 ± 0.18	83.91 ± 0.22	/
2N	Erasing × 1	82.46 ± 0.39	79.84 ± 0.13	88.35 ± 0.19	90.57 ± 0.21	83.91 ± 0.22	0.01
	Noise × 1	82.92 ± 0.39	80.52 ± 0.26	88.6 ± 0.31	91.34 ± 0.19	84.6 ± 0.13	0.58
	TSW × 1	84.55 ± 0.53	81.43 ± 0.76	89.54 ± 0.3	91.06 ± 0.26	85.79 ± 0.92	1.46
	DSM × 1	84.65 ± 0.13	81.59 ± 0.52	89.67 ± 0.28	91.52 ± 0.31	85.7 ± 0.76	1.61
4N	Erasing × 3	82.49 ± 0.37	79.84 ± 0.13	88.35 ± 0.19	90.58 ± 0.2	83.91 ± 0.22	0.02
	Noise × 3	83.2 ± 0.48	81.03 ± 0.44	88.62 ± 0.29	91.89 ± 0.11	84.95 ± 0.26	0.92
	TSW × 3	85.82 ± 0.48	82.43 ± 0.27	90.62 ± 0.35	91.86 ± 0.43	86.96 ± 0.58	2.52
	DSM × 3	85.85 ± 0.5	82.87 ± 0.42	90.58 ± 0.45	92.91 ± 0.26	87.35 ± 0.37	2.89

N, the data amount of the original training sample; 2N, the sample size after once augmentation; 4N, the sample size after three times augmentation; DA, data augmentation; None, original data; Erasing, random erasing; Noise, random noise; TSW, two sample weighting; DSM, difference of spectral mean; BLACK, Black peanut; DBS, Dabaisha; QC, Qicai; SLH, Silihong; XBS, Xiaobaisha; AIA, the average improved accuracy.

**Table 6:** Results of SVM training using all gradient data.

Sample Size	DA Method	BLACK (%)	DBS (%)	QC (%)	SLH (%)	XBS (%)	AIA (%)
N	None	87.1 ± 0.59	85.43 ± 0.58	94.54 ± 0.5	88.01 ± 0.64	84.39 ± 0.41	/
2N	Erasing × 1	86.69 ± 0.74	85.27 ± 0.48	94.5 ± 0.48	85.82 ± 0.47	83.31 ± 0.44	−0.78
	Noise × 1	87.06 ± 0.57	85.41 ± 0.58	94.39 ± 0.44	87.31 ± 0.57	84.07 ± 0.26	−0.25
	TSW × 1	89.01 ± 0.6	86.05 ± 0.45	95.31 ± 0.32	89.25 ± 0.51	86.02 ± 0.44	1.23
	DSM × 1	89.0 ± 0.86	86.71 ± 0.25	94.91 ± 0.3	91.11 ± 0.4	86.36 ± 0.17	1.72
4N	Erasing × 3	86.69 ± 0.7	85.32 ± 0.52	94.54 ± 0.44	85.7 ± 0.56	83.31 ± 0.37	−0.78
	Noise × 3	87.5 ± 0.68	85.19 ± 0.5	94.23 ± 0.45	88.4 ± 0.58	84.76 ± 0.23	0.12
	TSW × 3	90.69 ± 0.24	86.89 ± 0.43	95.53 ± 0.38	90.99 ± 0.72	87.13 ± 0.27	2.35
	DSM × 3	90.41 ± 0.4	87.46 ± 0.53	95.45 ± 0.25	93.07 ± 0.47	88.17 ± 0.93	3.02

N, the data amount of the original training sample; 2N, the sample size after once augmentation; 4N, the sample size after three times augmentation; DA, data augmentation; None, original data; Erasing, random erasing; Noise, random noise; TSW, two sample weighting; DSM, difference of spectral mean; BLACK, Black peanut; DBS, Dabaisha; QC, Qicai; SLH, Silihong; XBS, Xiaobaisha; AIA, the average improved accuracy.

**Table 7:** Results of MobileViT-xs training using all gradient data.

Sample Size	DA Method	BLACK (%)	DBS (%)	QC (%)	SLH (%)	XBS (%)	AIA (%)
N	None	90.3 ± 2.56	90.03 ± 1.55	92.48 ± 1.03	93.67 ± 0.78	88.12 ± 3.19	/
2N	Erasing × 1	95.07 ± 0.76	92.45 ± 1.29	97.33 ± 0.25	95.54 ± 0.22	96.9 ± 0.37	4.54
	Noise × 1	92.22 ± 1.16	91.92 ± 1.65	95.27 ± 1.49	94.57 ± 0.49	96.84 ± 0.17	3.24
	Rotation × 1	95.79 ± 0.57	92.13 ± 2.02	97.87 ± 0.45	95.99 ± 0.72	97.04 ± 1.09	4.84
	DSM × 1	96.16 ± 0.66	92.57 ± 1.35	97.95 ± 0.31	96.1 ± 0.57	97.04 ± 0.66	5.04

N, the data amount of the original training sample; 2N, the sample size after one augmentation; DA, data augmentation; None, original data; Erasing, random erasing; Noise, random noise; Rotation, rotation; DSM, a difference of spectral mean; BLACK, Black peanut; DBS, Dabaisha; QC, Qicai; SLH, Silihong; XBS, Xiaobaisha; AIA, the average improved accuracy.

## Data Availability

Data is contained within the article.
